# Letter to the editor: [^18^F]SMBT-1 PET quantification – methodological considerations and future directions

**DOI:** 10.1007/s12149-026-02203-2

**Published:** 2026-04-17

**Authors:** Qing Song, Mengxing Li

**Affiliations:** College of Acupuncture, Moxibustion and Tuina, Anhui University of Chinese Medicine, Hefei, China

Dear Editor,

We read with interest the article by Kotaro Hiraoka et al. titled *“Kinetic and quantitative analysis of [*^*18*^*F]SMBT-1 PET imaging for monoamine oxidase B”* published in *Annals of Nuclear Medicine* [1]. The authors address a critical step toward clinical translation: establishing a simplified quantification method without arterial sampling. While the study is well-conducted, several methodological aspects warrant further discussion to facilitate broader clinical application.

Firstly, A critical consideration for clinical application is the validation of reference regions in disease states. The authors demonstrate that SUVR-1 at 50–90 min correlates strongly with postmortem MAO-B distribution (*r* = 0.9362–0.9399) across multiple reference regions, including cerebellar cortex and white matter. However, in neurodegenerative conditions such as Alzheimer’s disease or progressive supranuclear palsy, these regions may themselves exhibit reactive astrogliosis and MAO-B upregulation [2, 3]. I would suggest that future studies systematically evaluate candidate reference regions in biomarker-confirmed patient cohorts to identify which remain stable across the disease spectrum. Cerebellar gray matter is often spared, but this is not universal—direct validation using arterial input function in patients would clarify whether SUVR-1 retains its accuracy when reference regions are compromised.

Secondly, the kinetic analysis relies on only six healthy subjects with complete arterial data. While sufficient for initial validation, this small sample raises questions about whether the optimal SUVR window (50–90 min) remains valid in patients with altered pharmacokinetics—those with blood-brain barrier disruption, reduced cerebral blood flow, or variable peripheral metabolism. I recommend replication in patient populations with confirmed neuroinflammation, including test-retest reliability assessments to determine minimal detectable change. Such data are essential for powering future therapeutic trials and understanding whether simplified measures capture treatment effects.

Furthermore, the possibility of further abbreviating the imaging protocol merits exploration. Given the strong correlations at 50–90 min, could scan duration be shortened? A 30-minute acquisition (e.g., 50–80 min) would improve tolerability for elderly or cognitively impaired patients [4]. Pharmacokinetic simulations or split-half analyses of existing data could identify the minimal scan duration preserving correlation with Vt. Additionally, as this tracer moves toward broader use, multi-center standardization of acquisition protocols and SUVR calculation methods will be critical for harmonizing data across sites.

This study significantly advances [¹⁸F]SMBT-1 toward clinical application. Neuroinflammation is increasingly recognized as a therapeutic target, and non-invasive MAO-B quantification could serve diagnostic, pharmacodynamic, and prognostic roles. We congratulate the authors on this encouraging work on kinetic and quantitative analysis of [¹⁸F]SMBT-1 PET imaging, and hope that these comments contribute to the ongoing discussion on optimizing quantification methods for clinical translation of MAO-B neuroimaging.
